# Buying Food on Sale: A Mixed Methods Study With Shoppers at an Urban Supermarket, Philadelphia, Pennsylvania, 2010–2012

**DOI:** 10.5888/pcd11.140174

**Published:** 2014-09-04

**Authors:** Etienne J. Phipps, Shiriki K. Kumanyika, Shana D. Stites, S. Brook Singletary, Clarissa Cooblall, Katherine Isselmann DiSantis

**Affiliations:** Author Affiliations: Shiriki K. Kumanyika, University of Pennsylvania Perelman School of Medicine, Philadelphia, Pennsylvania; Shana D. Stites, S. Brook Singletary, Clarissa Cooblall, Einstein Healthcare Network, Philadelphia, Pennsylvania; Katherine Isselmann DiSantis, Arcadia University, Glenside, Pennsylvania.

## Abstract

**Introduction:**

The obesity epidemic has drawn attention to food marketing practices that may increase the likelihood of caloric overconsumption and weight gain. We explored the associations of discounted prices on supermarket purchases of selected high-calorie foods (HCF) and more healthful, low-calorie foods (LCF) by a demographic group at high risk of obesity.

**Methods:**

Our mixed methods design used electronic supermarket purchase data from 82 low-income (primarily African American female) shoppers for households with children and qualitative data from focus groups with demographically similar shoppers.

**Results:**

In analyses of 6,493 food purchase transactions over 65 weeks, the odds of buying foods on sale versus at full price were higher for grain-based snacks, sweet snacks, and sugar-sweetened beverages (odds ratios: 6.6, 5.9, and 2.6, respectively; all *P* < .001) but not for savory snacks. The odds of buying foods on sale versus full price were not higher for any of any of the LCF (*P* ≥ .07). Without controlling for quantities purchased, we found that spending increased as percentage saved from the full price increased for all HCF and for fruits and vegetables (*P* ≤ .002). Focus group participants emphasized the lure of sale items and took advantage of sales to stock up.

**Conclusion:**

Strategies that shift supermarket sales promotions from price reductions for HCF to price reductions for LCF might help prevent obesity by decreasing purchases of HCF.

## Introduction

Research on food marketing practices has become a major focus of public health research 1) to identify practices that contribute to obesity or other risks for diet-related chronic diseases and 2) to inform the development of policies to change such practices (1–3). Food prices are a marketing variable of interest as they are among the most important influences on how people shop and what foods they buy (4–6). Research on food price effects has traditionally focused on regular prices, presumably because sale prices were considered transitory and, therefore, atypical (7). However, coupons and other temporary price reductions have become sufficiently commonplace in food retailing to be of interest in their own right (2,8,9). This change in pricing practices raises concern from a public health perspective because discounts do increase amounts purchased (2,8,9) and are more commonly applied to high-calorie foods and beverages than to healthful foods such as fruits and vegetables (LM Powell, PhD, University of Illinois at Chicago, written communication, January 2014; 10).

We analyzed supermarket food purchase data from a sample of primarily low-income African American female shoppers with children. Obesity prevalence is substantially higher among African Americans (children and adults) than among whites (11). Low-income consumers are more sensitive to food prices than are those with higher incomes (5). The main study objective was to determine whether the influence of sale prices on purchases of high-calorie foods differs from their influence on purchases of low-calorie foods. In this article, we use the terms “discount,” “sale,” and “sale price” interchangeably to refer to advertised price reductions from full (regular) prices.

## Methods

### Study design

We used a mixed methods quantitative–qualitative design (12): a longitudinal analysis of supermarket transactions for customers of a supermarket in Philadelphia, Pennsylvania, followed by a complementary study involving focus groups with shoppers from the same store. This design allowed us to use the quantitative data to inform development of focus group questions and then to use focus group findings to inform interpretation of the quantitative data. The supermarket, a large full-service store, is in and draws its customers primarily from a census tract with a racial composition that is 89.1% African American, 6.6% white, 4.3% other races, and 3% Latino (of any race) (13). Data analyses were conducted from October 2012 through November 2013. All procedures involving human subjects were approved by the institutional review board of the Einstein Healthcare Network.

### Supermarket purchase data

#### Data sources

Purchase data were drawn from baseline data collected in 2 studies of financial incentives for the purchase of fruits and vegetables before any intervention took place: a pilot study conducted in April 2010 through August 2010 (study 1; n = 30) (14), and a full-scale study conducted in December 2010 through October 2011 (study 2; n = 58) (15). Six people who participated in both studies were counted only in study 2. Both studies recruited convenience samples by advertisement at the supermarket. Shoppers were eligible if they identified themselves as the main household shopper and had at least 1 child under age 18 years at home. For data collection purposes, participants needed to have a no-cost shopper loyalty card for the study store. Following informed consent from each study participant, we obtained electronic records of their purchase data from the store analyst, for a period of at least 4 (study 1) or 8 (study 2) weeks prior. Electronic data about which of our food categories of interest were on sale versus sold at full price each week during periods covered by our purchase data were also provided.

#### Data set coding and food categorization

We examined all purchases in the data files to determine which food purchases fit in 1 of 7 study categories (4 high-calorie foods [HCF] and 3 healthful low-calorie foods [LCF]). Our coding scheme for identifying HCF and LCF, adapted from a study of home food environments (JE Holsten, unpublished doctoral dissertation, University of Pennsylvania, 2010), was based on evidence about foods and beverages associated with excess weight gain. Foods were categorized based on their energy density (kilocalories [kcal]/100 g) and other aspects of nutritional quality (16). Fruits, vegetables, and low-fat dairy foods were designated as LCF if they had less than 100 kcal/100 g. HCF were sweet, savory, and grain-based snack foods with 250 kcal/100 g or more. The HCF category also included sugar-sweetened beverages (SSBs): nondairy beverages with more than 10 calories per serving, excluding 100% fruit juice. Foods in other categories (eg, meats) were not analyzed (Appendix).

Quantity information was extracted using detailed descriptions contained in the participants’ purchase data files. For foods that did not have a specific volume or weight given in the store data, we used public sources of product information from manufactures, the US Department of Agriculture (17), and other product reference sources.

Types of sales promotions analyzed included electronic coupons (discounts redeemed at the point of sale using a loyalty card) for free items, mix-and-match store discounts (discounts for purchasing a set of items from the same manufacturer), and multi-purchase offers. Additional store discounts included price reductions on produce (per pound) and items on quick sale. Price reductions through paper coupons (4% of all discounts observed) usually could not be associated with a specific food product and were not analyzed.

The resulting purchase data were 16,638 purchases of which 1,172 were nonfood items, 61 for which only the brand could be identified, and 7,289 that were not in 1 of the 7 study categories. Of the remaining 8,116 items, 1,623 did not meet the HCF or LCF criteria for the relevant category (eg, dried fruits were excluded from the fruit category). The 6,493 food purchases covered 924 shopping occasions over 65 weeks and involved 2,223 price discounts.

#### Statistical analysis

Descriptive statistics were used to report participant characteristics for the full sample of 82 households. The samples from the 2 studies were compared by using unpaired *t* tests, Fisher’s exact tests, and Mann–Whitney tests.

For each food category and for LCF and HCF combined, we tabulated the percentage of purchases that involved discounts by food category and by unique shopper. We also calculated the mean percentage of weeks during the study periods that foods were offered on sale.

Confidence intervals (CIs) were bias corrected and accelerated with estimates based on 1,000 bootstrap samples. For each shopper, purchases of specific products were coded according to whether they were purchased on sale or at full price, linking date of purchase to the weeks that those products were offered on sale.

We used fixed effects logistic regression to estimate the ratio of the odds that a food was purchased when it was on sale compared with the odds that it was purchased at full price. For these analyses, CIs were constructed from robust standard errors. To assess how much shoppers saved from discounts, we estimated the mean percentage of discount savings (full price minus price paid then divided by full price) per shopping day. We used bivariate and multivariate fixed effects generalized linear models with a gaussian distribution and log links to assess the mean change in shopper spending that was related to increases of $1 in discount savings. To control for changes in amount spent by a shopper related to differences in the amounts of foods purchased, we used multivariate models to adjust statistically for quantities purchased on a given shopping day. In analyses of purchases in each study category except SSBs, we used number of ounces (weight) purchased as a covariate in the models to adjust for quantities of food purchased. For purchases of SSBs, we adjusted for number of fluid ounces (volume). These fixed effects analyses controlled for repeated observations of shoppers.

All statistical models controlled for exposure time. Estimates from models with log links were exponentiated. All statistical tests were 2-sided. *P* values of .05 or less were considered significant. Statistical analyses were performed by using Stata 12 (Stata Corp LP), SAS 9.3 (SAS Institute Inc), and PASW 18.0 (PASW Inc).

### Qualitative data

We recruited focus group participants by using flyers placed in supermarket bags at the study store. Adult caregivers with at least 1 child under age 18 years living in the household were eligible to participate. Interested participants contacted the research office, and if eligible, were informed about the purpose of the focus group. Informed consent was obtained over the telephone, after which we obtained basic sociodemographic information about the shopper and his or her household.

A moderator guide (based on results of the quantitative analyses) was developed to elicit information about shopping practices and patterns of buying on sale (Appendix). Three focus groups were conducted in an upstairs room at the study store in May and June 2013. Sessions were approximately 90 minutes and were digitally recorded. Participants received a $35 supermarket gift card.

Three members of the research team met to review the purpose of the focus groups and the process to be used in analyzing verbatim focus group transcripts. They separately read through and labeled the transcripts by highlighting key words, phrases, sentences, and chunks of related sequences of text and labeling those responses (18). Through team discussion, key issues and recurrent themes within and across groups were identified (12).

## Results

Of 82 household shoppers, most were women and identified themselves as African American. Almost one-third of participants reported an annual household income of less than $15,000; more than half were enrolled in the Supplemental Nutrition Assistance Program (SNAP). More than half had education beyond a high school diploma. Study 1 participants had more children in the household and were younger than study 2 participants (Table 1).

**Table 1:** Demographic Characteristics of Supermarket Shoppers, Philadelphia, Pennsylvania, April through August 2010 (Study 1) and December 2010 through October 2011 (Study 2)

Characteristic	Overall Sample (N = 82)	Study 1 (n = 24)^a^	Study 2 (n = 58)	*P* Value^b^
**Household size, mean (SD)**	4.0 (1.4)	4.4 (1.5)	3.8 (1.4)	.15
**Number of children in household, mean (SD)**	1.9 (1.1)	2.3 (1.2)	1.7 (1.0)	.05
**Age of respondent, y, mean (SD)**	47.5 (14.2)	40.5 (14.6)	50.4 (13.2)	.002
**Average child age, y, mean (SD)**	8.9 (4.7)	8.1 (4.9)	9.2 (4.6)	.31
**Sex, n (%)**				
Female	68 (82.9)	21 (87.5)	47 (81.0)	.75
Male	14 (17.1)	3 (12.5)	11 (19.0)	
**Race/ethnicity^c^, n (%)**				
African American	79 (96.3)	23 (95.8)	56 (96.6)	.51
Non-Hispanic white	2 (2.4)	1 (4.2)	1 (1.7)	
Hispanic	4 (4.9)	3 (12.5)	1 (1.7)	
**Marital status, n (%)**				
Married or living with partner	28 (34.1)	8 (33.3)	20 (34.5)	.62
Single or never married	28 (34.1)	10 (41.7)	18 (31.0)	
Divorced, separated, or widowed	26 (31.7)	6 (25.0)	20 (34.5)	
**Education, n (%)**				
≤High school diploma	37 (45.1)	11 (45.8)	26 (44.8)	.52
Some college or associate’s degree^d^	32 (39.0)	11 (45.8)	21 (36.2)	
≥College graduate	13 (15.9)	2 (8.3)	11 (19.0)	
**Annual household income, $, n (%)^e^**				
<15,000	25 (30.5)	11 (45.8)	14 (24.1)	.19
15,000–25,000	32 (39.0)	6 (25.0)	26 (44.8)	
25,001–50,000	20 (24.4)	6 (25.0)	14 (24.1)	
50,001–60,000	3 (3.7)	1 (4.2)	2 (3.4)	
**SNAP enrolled, n (%)**	47 (57.3)	11 (45.8)	36 (62.1)	.22
**WIC enrolled, n (%)**	23 (28.0)	6 (25.0)	17 (29.3)	.79
**WIC and SNAP enrolled, n (%)**	17 (20.7)	5 (20.8)	12 (20.7)	>.99

Abbreviations: SD, standard deviation; SNAP, Supplemental Nutrition Assistance Program; WIC, Supplemental Nutrition Program for Women, Infants, and Children.

a Six of the 30 households in study 1 enrolled in study 2. Data on dually enrolled households are included only in the study 2 column.

b *P* values based on χ^2^ tests (or Fisher’s exact tests) for categorical variables and bootstrapped *t* tests for continuous variables.

c Percentages do not sum to 100% because 4 respondents identified as both African American and Hispanic. One respondent chose not to answer.

d Category includes some college, associate’s degrees, and technical school degrees.

e Percentages do not sum to 100% because 2 respondents chose not to answer.

There was considerable overlap in the percentage of weeks that HCF and LCF were on sale (Figure). SSBs and savory snacks were on sale on average about one-third of the study period (35% and 32%, respectively). The other HCF and the LCF were on sale 15% to 23% of the time.

**Figure:**
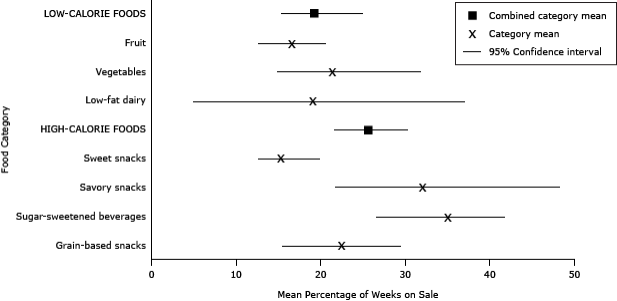
Mean percentage of weeks foods were on sale, by food category and aggregate food category, in an urban supermarket, Philadelphia, Pennsylvania, April through August 2010 and December 2010 through October 2012. **Table:** 

Food Category	Mean Percentage of Weeks on Sale (95% Confidence Interval)
**Low-calorie foods**	19.2 (15.4–25.1)
Fruit	16.5 (12.7–20.7)
Vegetables	21.4 (14.9–31.9)
Low-fat dairy	19.0 (5.0–37.1)
**High-calorie foods**	25.7 (21.6–30.4)
Sweet snacks	15.3 (12.6–20.0)
Savory snacks	32.0 (21.8–48.3)
Sugar-sweetened beverages	35.0 (26.6–41.9)
Grain-based snacks	22.5 (15.5–29.6)

All shoppers bought on sale at least 1 food included in the study. More than half made 30% or more of their purchases on sale. The proportion of purchases made on sale ranged from 3% to 75%.

Shoppers were more likely to purchase sweet snacks (odds ratio [OR], 5.9; 95% CI, 3.5–10.0), SSBs (OR, 2.6; 95% CI, 1.9–3.7), and grain-based snacks (OR, 6.6; 95% CI, 3.6–12.0) when those items were on sale compared with when sold at full price. The likelihood of buying LCF on sale versus at full price was not significant for fruits (OR, 1.1; 95% CI, 0.7–1.7) or vegetables (OR, 1.3; 95% CI, 0.9–1.8) (Table2).

**Table 2:** Odds of Purchasing Foods on Sale Versus Not on Sale Among Shoppers Who Purchased These Foods, Philadelphia, Pennsylvania, April through August 2010 and December 2010 through October 2012

Food Category	OR^a^ (95% CI^b^)	*P* value
**Low-calorie foods**	**1.3 (1.0–1.7)**	**.08**
Fruit	1.1 (0.7**–**1.7)	.61
Vegetables	1.3 (0.9**–**1.8)	.15
Low-fat dairy	4.7 (0.9**–**24.9)	.07
**High-calorie foods**	**2.4 (2.0–3.0)**	**<.001**
Sweet snacks	5.9 (3.5**–**10.0)	<.001
Savory snacks	1.1 (0.6**–**2.0)	.77
Sugar-sweetened beverages	2.6 (1.9**–**3.7)	<.001
Grain-based snacks	6.6 (3.6**–**12.0)	<.001

Abbreviations: OR, odds ratio; CI, confidence interval.

a Fixed effects logistic regression models predict that food was purchased (“1”) compared with not purchased (“0”) in weeks that food was on sale (“1”) compared with weeks food was sold at full price (“0”). Estimates are based on 79,087 observations from 81 households that had purchase data on more than 1 day. Models adjusted for household exposure time in the study.

b 95% CIs constructed from robust standard errors.

When shoppers bought foods on sale, the mean discount varied from 3.9% off the full price for sweet snacks to 37.9% for SSBs and 43.8% for low-fat dairy products (Table 3). On the basis of unadjusted analyses, shoppers spent more on average in a food category as the amount they saved in that category increased, particularly for savory snacks (*P* ≤ .002). Shopper spending on low-fat dairy products, which were infrequently purchased, was the singular exception (*P* = .60). Statistically adjusting for quantity purchased explained (ie, attenuated to nonsignificance) the increase in spending for vegetables, savory snacks, and grain-based snacks (*P* ≥ .55) but only partially explained the increase for SSBs, sweet snacks, and fruit (*P* ≤ .03).

**Table 3:** Mean Discount Savings and Percentage Change in Spending Associated With Increases in Discount Savings Among Supermarket Shoppers, Philadelphia, Pennsylvania, April through August 2010 and December 2010 through October 2012

Food Category	Mean Discount Savings^a^, % (95% CI)^c^	Mean Change in Spending, Unadjusted^a^		Mean Change in Spending, Adjusted^a^^,^ b	
		% (95% CI)^d^	*P* Value^e^	% (95% CI)^d^	*P* Value^e^
**Low-calorie foods**	**21.2 (18.6–24.2)**	**17.8 (10.2–21.7)**	**<.001**	**2.9 (0–4.6)**	**.08**
Fruit	4.3 (3.6**–**5.2)	12.7 (2.5–17.6)	.002	6.7 (0.9–11.3)	.03
Vegetables	22.6 (19.3**–**26.3)	12.7 (8.3–23.4)	<.001	−1.2 (−6.0 to 3.7)	.55
Low-fat dairy	43.8 (42.8–44.4)	5.8 (−8.9 to 15.0)	.60	−10.5 (−43.8 to 9.1)	.44
**High-calorie foods**	**23.5 (21.5–25.6)**	**9.5 (8.8–12.6)**	**<.001**	**3.3 (1.6–5.9)**	**.002**
Sweet snacks	3.9 (3.3**–**4.5)	9.9 (6.9**–**11.4)	<.001	5.2 (2.2–7.2)	<.001
Savory snacks	24.8 (22.8**–**26.8)	23.3 (11.7**–**28.2)	<.001	1.6 (−7.9 to 3.0)	.61
Sugar-sweetened beverages	37.9 (34.9**–**40.7)	11.4 (10.4**–**18.8)	<.001	5.3 (4.5–12.3)	.001
Grain-based snacks	20.9 (18.9**–**23.1)	9.1 (5.7**–**10.2)	<.001	−0.01 (−3.5 to 1.6)	.97

Abbreviation: CI, confidence interval.

a Spending is defined as the purchase price minus any discount savings and thus equal to the amount paid by the shopper, expressed as a percentage of dollars spent on all foods in the category.

b Adjusted analyses control statistically for the number of ounces purchased.

c Savings is defined as the dollar amount by which the purchase price was reduced by discounts, expressed as a percentage of dollars spent on all foods in the category.

d All 95% CIs are bias corrected and accelerated with estimates based on 1,000 bootstrap samples.

e *P* values were obtained from fixed effects generalized linear models with gaussian distribution and log links.

The 3 focus groups had a total of 20 participants, all identified as African American; 90% were women. The mean age was 49.6 years (SD, 15.3; range, 18–74). Focus group participants were demographically similar to shoppers in the purchase data except that a smaller percentage were enrolled in Supplemental Nutrition Program for Women, Infants, and Children (5% in the focus group vs 28% of the shoppers with purchase data; *P* = .04).

The importance of buying on sale was the main organizing theme voiced by focus group participants (Box). They described skills used to identify and select purchases. They expressed pride in the time, effort, and proficiency they used to identify sale items. Many cited supermarket circulars as important sources for finding which items were on sale and comparing sales across markets. They described the value of using sales to stock up on items and had plans for how to maximize use of larger quantities purchased. Some participants who used shopping lists described deviating from them to take advantage of sales. Participants spoke about the perceived expense of more healthful foods such as fresh produce and how price factored into their decisions to buy them. Taking advantage of sales was specifically mentioned as a strategy for being able to afford fresh produce and salads.

**Box. Focus Group Quotes About Supermarket Purchasing and Expense of Eating Healthy**

**Importance of buying on sale**

So I will buy, if it’s cheap, it’s on sale, and even if I don’t need it, I’ll still buy it. [Focus group 2]

**Circulars as source of information about sales**

I always use the circulars and very rarely do I buy anything that’s not on sale, especially if it’s a nonnecessity item. [Focus group 1]

**Importance of stocking up**

I think if it’s something on sale that you need, or that you know you’re going to get to, or you might run out of, and it’s on sale, stock up on that. [Focus group 2]

**Comparison shopping**

It’s hard, I mean, if we didn’t actually make it a job to comparison shop, we would be going home with one bag of food for the week. But we really, diligently, compare and it takes two of us to do it. [Focus group 1]

**Maximizing use of larger quantities purchased**

And this is how we gain, it’s not that we don’t need, you always have to have ideas of you could do with what you have. [Focus Group 1]

Well it’s important but if you have a freezer, another freezer besides your refrigerator, that’s when you do that [purchase multiple items]. [Focus Group 3]

You know what I mean? ’Cause I know [my son] likes the high brand, but I mix it in with the cheaper brand. Sometimes he eats this and sometimes he eats that. [Focus Group 3]

**Use of lists**

Limited users:

Like I have a list and come into the store and I’ll say I’m gonna get all the things on my list, but then when I walk around and see the sales, I can get it. . . . So I’m gonna get what I got on the list and get that sale. And try to get one or two, more than one, you know? [Focus group 3]

Nonusers:

I don’t come in here with a slip. Whatever’s on sale, that’s what I’m going to get. [Focus group 1]

Users:

I try to shop with a list because when I shop with a list it’s, it’s more organized. I already know what I have in my cabinet, and so I’m not double dipping, double buying, um, so I do like to shop with a list. [Focus group 3]

**Expense of eating healthfully**

Sometimes they have produce marked down, salad ninety-nine cents, I’ll eat salad all day. So that’s how I shop. I look for fruits, when it’s on sale, I just buy it. So if that week, if that food is not on sale, I’m not going to lose out because I already had it. So that’s how I shop. [Focus group 1]

They would do better if they would mark down the vegetables more because it’s . . . good for you to eat more vegetables. [Focus group 1]

If I see spinach on sale, I'm buyin’ it. I don't see it often. [Focus group 1]

Vegetables, they’re so expensive. . . . It’s like you have to forgo something else, to get some fresh vegetables. [Focus group 1]

Because it’s good for you, anything that’s good for you, it costs a fortune, so you have to make a choice. [Focus group 1]

Yesterday I was in a terrible predicament in the store, like do I buy the soap powder and bleach, or do I buy the fruit and things that I know my grandkids need. [Focus group 2]

## Discussion

The quantitative and qualitative data were congruent in indicating that shoppers in our sample sought out and took advantage of discounts. This finding is not surprising given evidence that low-income shoppers are generally price sensitive (5), need to stretch food dollars in order meet other needs (19), and use specific price-searching strategies (6,20). We noted an increased likelihood of buying 3 of the 4 HCF on sale. This effect was not observed for LCF. However, an effect of sale prices on LCF and HCF was observed with respect to amounts spent. Specifically, spending increased as the size of the discount increased for 2 of 3 LCF categories (except low-fat dairy products, which were seldom purchased overall) and all HCF categories.

One explanation for the observed increase in the likelihood for purchasing HCF on sale versus full price might be that HCF were on sale more frequently than LCF and thus more likely to be purchased during a sales period. However, this hypothesis was not confirmed by our data on sweet and grain-based snacks. Sweet snacks, for example, were on sale the least often of any food category and yet were 5 times more likely to be purchased on sale versus at full price. It is possible that the lure to purchase sweet snacks when they were on sale was heightened because sales were less frequent.

The applicability of these results to low-income and African American shoppers adds to the importance of our findings. Consumption patterns of foods in our HCF and LCF categories do not meet dietary guidelines for the US population in general, and the dietary patterns of African American and low-income adults and children are not as healthful as the dietary patterns of whites (21,22). For example, African Americans consume more SSBs than whites do and fewer fruits, vegetables, and low-fat dairy products (22).

Strategic use of price discounts or subsidies has been suggested as a policy approach for increasing purchases of fruits and vegetables and other healthful foods (15,23–26). Focus group data suggest that shoppers want to see more sales on fruits and vegetables. Yet purchase data suggest that the response to sales on fruits and vegetables was to spend more (through purchasing larger quantities or more costly varieties that became affordable when on sale) rather than to increase purchase frequency. Our study found that the effect of reducing price on purchasing behavior was different for fruits and vegetables than for HCF. This finding might be due to the qualitative differences between the HCF and LCF we included in our study. HCF tend to have longer shelf lives, more familiar branding, and preferred taste qualities (eg, salt, fat), all of which encourage purchasing in large quantities and quicker consumption (8,27). The value of the discount for fruits and vegetables may have been an additional factor in shoppers’ decision about whether to purchase them. Randomized trials suggest that discounting fruits and vegetables works best when the discount is large (eg, 50%) (14,23,24,28), larger than the percentage savings we observed, especially for fruit.

Various taxation strategies have been studied as a way of raising prices to discourage consumption of HCF (29). Although potentially effective, taxation strategies may be sufficiently unpopular to be feasible for wide adoption. Discounts are clearly popular. However, whereas taxes raise revenue, the use of discounts may come at a cost, to government, retailers, or manufacturers who supply retailers. Nevertheless, given the frequency of price reductions on HCF as observed here and elsewhere, the feasibility and impact of decreasing the frequency of these reductions is worth exploring. The costs and health benefits of redirecting sales promotions to stimulate purchases of more healthful products are also worth exploring. To our knowledge, the effects of limiting price reductions (in frequency or size of the discount offered) on HCF on purchasing behaviors while increasing their use on LCF has not been studied.

Strengths of our study include the objective, quantitative data from shoppers at a single supermarket, collected over a period that included all 4 seasons, with multiple weeks of purchase data for each shopper. Using data from a single supermarket facilitates attribution of observed effects to the discounting patterns rather than to other retailing variables, which were presumably the same for all shoppers during any given week. Having complementary qualitative data for shoppers from the same store added context to the findings. We focused on a high-risk population and on specific foods in well-defined categories associated with obesity risk. Because we did not have information that would allow us to interview focus group participants while they were actually shopping, a limitation is our inability to tease apart how the sale influenced purchases in terms of factors such as size of discount or promotional format. We also cannot assess the effect that buying sale products had on eating behaviors or overall dietary quality for shoppers or other household members.

Our findings bring a new dimension to studies of how food prices can influence obesity development in high-risk groups. Research focused specifically on the types of foods offered through sales and associated promotional strategies may identify new causal pathways for an influence of food prices on excess weight gain or on the difficulty of losing weight among those already overweight or obese. The feasibility of retailing policies and practices that discourage discounts on HCF should be explored.

## Appendix

This file is available for download as a Microsoft Word document.
