# Supplementary material for: Buying Food on Sale: A Mixed Methods Study With Shoppers at an Urban Supermarket, Philadelphia, Pennsylvania, 2010–2012
Source: Prev Chronic Dis. 2014 Sep 4;11:E151. doi: 10.5888/pcd11.140174 (PMC4157594; doi:10.5888/pcd11.140174)
Supplement: Supplementary file 1 [file 14_0174_01.doc]

**Buying Foods on Sale: A Mixed Methods Study with Shoppers at an Urban Supermarket**

**APPENDIX**

**Appendix.** Food category definitions and examples of items included or excluded by the coding rules for each group^a^

| **Category** | **Food Category Definition** | **Examples of included Food and Drink Items** | **Examples of excluded Food and Drink Items** |
| --- | --- | --- | --- |
| Fruit | Any fruit based item - fresh, canned, frozen, dried, whole, cut-up, pureed, or 100% juice | Apples, strawberries, peaches, 100% fruit juices | Fruit items with >100 kcal/100g:  avocados, jams, raisins |
| Vegetable | Any vegetable based item - raw, cooked, fresh, frozen, canned, dried, whole, cut-up, pureed, mashed varieties, legumes, or 100% juice | Peppers, broccoli, tomatoes, green peas, white potatoes, canned tomatoes, dry navy beans | Vegetable items with >100 kcal/100g:  baked beans, potato salad, sweet potatoes |
| Low-fat dairy | Foods made from milk that retain their calcium content with < 1% milk-fat. Foods made from milk with little to no calcium, such as cream cheese, cream, and butter, are not included. | Non-fat or 1% milk, yogurt, cottage cheese | Low-fat dairy items with >100 kcal/100g:  fat-free or low-fat cheese, Milk based-desserts |
| Sweet snacks | High-energy food items consumed outside a meal with a sweet taste - baked goods, ready-to-make/ingredients, frozen treats, milk-based desserts, candy, sweet toppings; > 225 kcal/100g. | Cookies, muffins, cake/brownie mixes, ice cream, frozen yogurt, jelly, chocolate sauce, chocolate chips, granola bars | Sweet snack items with < 225 kcal/100g:  sorbet, Jell-O^TM^, fat-free pudding |
| Savory snacks | High-energy food items consumed outside a meal with a savory or salty taste; > 225 kcal/100g. | Potato chips, pretzels, beef jerky, crackers, fried vegetables, nuts | Savory snack items with <225/100g (no examples identified in this sample) |
| Sugar-sweetened beverages^b^ | Any non-dairy beverage with >10 calories per serving excluding 100% fruit juice | Soda, sports drinks, fruit punch |  |
| Grain-based snacks^c^ | Grain-based foods sometimes eaten as snacks; > 225 kcal/100g | Sweetened cereals, pizza, frozen hot/lean pockets, burritos, bagel bites, poptarts/toaster strudel | Non-sweetened hot cereals and ready-to-eat cereals with <225/100g |
| ^a^ Adapted from Holsten, Joanna E. "Exploring the Relationship between Middle School Children’s Body Mass Index and the Home Food Environment within the Contextual Process of Food Choice." (JE Holsten, Ph.D. dissertation, 2010). Caloric cut-offs were adjusted for these analyses to clearly differentiate between low- and high-energy foods based on the World Cancer Research Fund Guidelines (World Cancer Research Fund. Energy density: finding the balance for cancer prevention. http://www.wcrf-uk.org/PDFs/EnergyDensity.pdf. Updated 2012. Accessed June 5, 2014.).  ^b^ Energy-density is not applied to liquids that are mostly water.  ^c^ This category was added (not in original categories by Holsten (2010)) because there was a high frequency of grain-based items which would not be coded.  KEY: kcal – kilocalories g – gram | | | |

| **Appendix**. Focus Group Moderator Guide |
| --- |
| **Introduction and Purpose**  Thank everyone for coming  Describe the focus of the study, length of time for group  Describe process, audio recording, confidentiality  Emphasize interest in hearing everyone’s views  Ask individuals to introduce self  **Topic – Question – Follow-up and Probes**   1. **Topic: Use of Grocery Shopping Lists**   **Question:** Do any of you make a grocery list before you go to the store?  **Follow-up:** For those who use a grocery list  - Why do you use a list? Do you ever buy items that are not on your list?  **Follow-up:** For those who do not have a written list   - What is your strategy for food shopping?   ***Probe*:** Responses to understand use of list; how is list used; helpful vs. not  ***Probe*:** What specific kinds of products are purchased?  ***Probe*:** Impact of price promotions on purchasers with lists and without lists   1. **Topic: Importance of discount savings**   **Question:** How much do you rely on discount savings to meet your shopping needs?  **Follow-up:** What kinds of deals or price promotions are important to you?  **Follow-up:** What kinds of foods do you usually get with discounts?  **Follow-up:** Are there foods that you would like to see on sale more often?  ***Probe*:** Responses related to importance of savings  ***Probe*:** Specific purchases bought on sale or desired to be on sale, e.g., meat and poultry, fish, dairy products, bread, breakfast foods, fruits and vegetables, fruit juices, soft drinks   1. **Topic: Source of information about supermarket sales**   **Question:** How do you find out about the store deals or discounts?  **Follow-up:** Do you read anything before going to the store?  **Follow-up:** Do you go online?  **Follow-up:** What do you look for in terms of discounts?  ***Probe*:** Understand how participants find out about discounts  ***Probe*:** Understand how they share information.   1. **Topic: Buying patterns, i.e., bulk buying**   **Question:** A lot of people buy multiples of certain items. Does anyone do this?  **Follow-up:** What influences the quantities of products that you buy?   - Understand about buying the larger sizes/packs of items, as well as buying multiples   ***Probe*:** Understand whether buying a large amount change the way they shop the next time. Does what they buy last a long time? etc.  ***Probe*:** Can anyone name a situation in which you would decide NOT to buy a larger quantity even when it looks like a good deal? etc.  ***Probe*:** Understand specific products   - Are there issues related to not being able to carry products home, storage space, spoilage or waste?   ***Probe*:** What kinds of things would you buy in quantities?  ***Probe*:** How often is this related to the price promotions?  ***Probe*:** What wouldn’t you buy in quantities? Why?  **Closing**  Ask if anyone has any final comments related to the discussion  Thank participants and provide gift card |
